# Role of estrogen in treatment of female depression

**DOI:** 10.18632/aging.205507

**Published:** 2024-02-02

**Authors:** Qihan Sun, Guangquan Li, Fangyi Zhao, Mengmeng Dong, Wei Xie, Qianqian Liu, Wei Yang, Ranji Cui

**Affiliations:** 1 Jilin Provincial Key Laboratory on Molecular and Chemical Genetic, The Second Hospital of Jilin University, Changchun, Jilin 130000, P.R. China; 2 Department of Neurology, The Second Hospital of Jilin University, Changchun, Jilin 130041, P.R. China

**Keywords:** depression, estrogen, HPA axis, inflammation, synaptic plasticity

## Abstract

Depression is a neurological disorder that profoundly affects human physical and mental health, resulting in various changes in the central nervous system. Despite several prominent hypotheses, such as the monoaminergic theory, hypothalamic-pituitary-adrenal (HPA) axis theory, neuroinflammation, and neuroplasticity, the current understanding of depression’s pathogenesis remains incomplete. Importantly, depression is a gender-dimorphic disorder, with women exhibiting higher incidence rates than men. Given estrogen's pivotal role in the menstrual cycle, it is reasonable to postulate that its fluctuating levels could contribute to the pathogenesis of depression. Estrogen acts by binding to a diversity of receptors, which are widely distributed in the central nervous system. An abundance of research has established that estrogen and its receptors play a crucial role in depression, spanning pathogenesis and treatment. In this comprehensive review, we provide an in-depth analysis of the fundamental role of estrogen and its receptors in depression, with a focus on neuroinflammation, neuroendocrinology, and neuroplasticity. Furthermore, we discuss potential mechanisms underlying the therapeutic effects of estrogen in the treatment of depression, which may pave the way for new antidepressant drug development and alternative treatment options.

## INTRODUCTION

Depression is a prevalent mental illness characterized by low mood, lack of pleasure, and cognitive decline [1]. The incidence is closely associated with symptom severity and obstacles [2]. Epidemiological surveys have reported that the twelve-month and lifetime prevalence of major depression exceeds 10% and 20%, respectively, with over 39% of patients exhibiting suicidal tendencies [3]. Notably, there are significant gender differences in the incidence of depression [4], Linnéa Nöbbelin et al. described a significantly higher incidence of depression and Major depressive disorder (MDD) in women [5]. For a cross-sectional trial aimed at the general population in China, Wang et al. found that the incidence rate of depression in women is three times that in men [6, 7]. For American teenagers, the incidence rate of depression is about 25% for females and about 10% for males [8]. Studies have also found that women are dominant in depression, anxiety, and neurocognitive disorders [9]. The underlying reasons for women’s susceptibility to depression remain unclear, but the fluctuating stages of sex hormones during a woman’s life cycle, such as before and after pregnancy and perimenopause, are often considered susceptible periods for female depression [10]. These evidences suggest that sex hormones may play a crucial role in the biological risk factors for depression. Further research is needed to elucidate the underlying mechanisms of sex hormones in depression and develop targeted interventions that may benefit female patients with depression.

Depression is a complex neurological disorder with a multifaceted pathogenesis, involving changes in the central nervous system. Part of the risk of depression is mediated by environmental and genetic factors [11], and although its mechanism is currently unclear, the interaction of multiple factors, including early life events, social stress, health, and medication, seems to be the foundation of its development [12]. Although several hypotheses have been proposed, including the monoaminergic hypothesis, the HPA axis hypothesis, the inflammation hypothesis, and the neuroplasticity hypothesis, the underlying mechanisms of depression remain unclear. Among these hypotheses, the monoaminergic hypothesis is the most widely used in clinical treatment, which proposes that a decrease in synaptic monoamine transmitters such as serotonin, dopamine, and norepinephrine is the main cause of depression. The current treatment methods mostly revolve around the increase of monoamine transmitters [13]. However, this treatment method is considered to have certain limitations. At least half of the patients report that the effect is not satisfactory, and the clinical response only takes effect after long-term treatment [14]. The HPA axis hypothesis posits that stress activates the HPA axis, leading to excess corticosterone (CORT) exposure in the brain, while the inflammation hypothesis suggests that mood disturbances often accompany an inflammatory response [15]. Furthermore, the neuroplasticity hypothesis emphasizes the importance of synaptic function and neurogenesis in various brain regions, particularly the hippocampus (HP), in depression pathogenesis [16]. Current research highlights the need to explore the underlying mechanisms of depression and develop novel therapeutic strategies.

Estrogen, an important steroid hormone secreted by the ovaries in the female body, exists in three forms, including estrone (E1), estradiol (E2), and estriol (E3) [17]. E2 is metabolized in the liver to produce E1 and E3. Although E3 is often considered to be inactive as a metabolite of E2 [18], some researchers have suggested that the ratio of E3 to progesterone may interact with depressive symptoms to predict preterm birth [19]. Understanding the roles of these different forms of estrogen is essential for elucidating the underlying mechanisms of their effects on various physiological and pathological processes. As the primary sex hormone secreted by the ovaries, E2 is recognized for its ability to modulate mood and cognitive functions, making it a key therapeutic target for depression. In fact, research has found that depressed women exhibit significantly lower levels of estrogen than healthy women [20]. Research has shown that there is a statistically significant correlation between low estrogen levels and the likelihood of developing depression in postmenopausal elderly women [21]. Furthermore, the removal of both ovaries has been established as a reliable model for inducing depression-like symptoms due to the resulting decrease in estrogen levels [22]. Stress, a well-established predictor of depression, has been shown to interact with estrogen to regulate both behavioral and biochemical changes associated with depression [23, 24]. Importantly, estrogen has been found to modulate neurotransmitter systems such as glutamate, gamma-aminobutyric acid (GABA), serotonin, and dopamine, highlighting the complex role of this hormone in the pathogenesis of depression [10, 25, 26], inhibits GABA and inhibits input [27], and the serotonergic and dopaminergic systems can also be regulated by estrogen [28–30]. Severe fluctuations in estrogen levels during periods of stress exposure can increase the likelihood of depression, anxiety, and post-traumatic stress disorder [17]. These findings have led to an increased interest in the potential role of estrogen in the pathogenesis of depression. Although estrogen is widely believed to have neuroprotective effects [31], research has shown that estrogen exceeding physiological doses can actually activate inhibitory estrogen receptor beta (ERβ) [32, 33], thereby exacerbating depressive like behavior [34]. Therefore, regulating the estrogen level curve and the activation of estrogen receptors may become potential targets for the treatment of female depression.

## Female depression

There is mounting evidence to suggest that fluctuations in estrogen levels in women can significantly influence the risk of developing depression [35]. This type of depression caused by physiological or pathological fluctuations in estrogen and progesterone levels is called female depression. From the onset of puberty to the cessation of menopause, women’s neurotransmitter systems are exposed to cycles of hormonal fluctuation that vary on a monthly or even age-related basis. Since the onset of puberty, girls have shown a significantly higher incidence of emotional disorders than boys. The changes in estrogen and progesterone levels during the luteal phase of the menstrual cycle can trigger a range of symptoms, including anxiety, depression, and insomnia, collectively known as Premenstrual Dysphoric Disorder (PMDD) [36]. Relief from these mood disorders is generally achieved with the onset of menstruation [37].

Women’s higher susceptibility to MDD has been associated with their strong fluctuations in sex hormone levels and increased sensitivity to sex hormones [38, 39]. During different reproductive stages, women are at risk of developing female depression such as perinatal depression (PND) and postpartum depression (PPD), due to the influence of large fluctuations in ovarian hormones [40]. Epidemiological and clinical studies have confirmed that approximately 10%-20% of women experience mental disorders during the perinatal period [41]. PND is typically considered an affective mental illness that occurs between conception and 12 months after delivery, while PPD is defined as anxiety, sleep disturbance, and loss of appetite that occurs during delivery to 4 weeks postpartum [42]. Large fluctuations in estrogen and progesterone during the perinatal period have been identified as an important source of stress for susceptible women, leading to the development of PPD symptoms that coincide with the cycle of ovarian hormone fluctuations [43, 44]. Moreover, studies have revealed that women with a history of PPD experience significant mood disorders after treatment and withdrawal of estrogen, whereas women without such a history do not exhibit similar symptoms despite being exposed to the same hormone environment [44].

When women enter the perimenopausal period, their ovarian function begins to decline, the menstrual cycle undergoes significant changes, and their susceptibility to depression also increases [45]. Studies have shown that women with a history of depression are at an increased risk of developing perimenopausal depression [46]. Depressive symptoms during hormonal fluctuations are often accompanied by disturbances in estrogen levels (Table 1), providing further evidence of estrogen’s impact on women’s susceptibility to depression.

**Table 1:** Summary of changes of E2 levels in different periods of female depression.

**Period**	**Condition**	**Estrogen level (compared to normal)**	**Symptom**	**Object**	**References**
Menstrual Period	Follicular phase	Decreasing	Depression	Women (20–49 years old)	[163]
	Early luteal	Decreasing	Premenstrual dysphoric disorder	Women	[164]
	Late luteal	Decreasing	Premenstrual dysphoric disorder	Women	[164]
Pregnancy	Week 36 of gestation	Decreasing	Postpartum depression	Women	[165]
Labour	Childbirth day	Unchanging	Postpartum depression	Women	[166, 167]
Postpartum	Day 3 postpartum	Increasing	Postpartum depression	Women	[168]
	Day 3 postpartum	Unchanging	Major depressive disorder	Women	[168]
	Day 5 postpartum	Increasing	Postpartum depression	Women	[167]
Perimenopause	Early Postmenopause	Decreasing	Perimenopausal depression	Women (54.4 ± 4.9 years old)	[169]

## Estrogen receptors and related signaling pathways

Estrogens are a group of steroid hormones, including E1, E2, and E3, that are synthesized and secreted by the ovaries. Among them, E2, with the molecular formula C_18_H_24_O_2_, is the most biologically active and abundant estrogen in the body, and it serves as a primary marker of gonadal function. E2 plays a crucial role in the development of female secondary sexual characteristics, as well as the regulation of the menstrual cycle and pregnancy. As previously mentioned, fluctuations in endogenous estrogen levels are pivotal in the pathogenesis of depression in women [47]. Rapid changes in E2 levels can heighten the susceptibility to depression in women [48, 49]. Furthermore, when the brain is exposed to low levels of E2, women must rely on multiple pathways to adapt to the lack of circulating estrogen to maintain central nervous system homeostasis [50].

E2, a crucial hormone in the female reproductive system, is present in two forms - α and β, with 17β-E2 being the more active form. Studies have demonstrated the potential of 17β-E2 in reducing depression-like behaviors [51]. In female rats, 17β-E2 was found to enhance neurogenesis [52]. *In vitro* experiments showed that 17β-E2 could potentially restore the loss of excitatory synapses caused by altered expression of Disrupted in Schizophrenia 1 (DISC1) [53]. Conversely, the biological function and mechanism of action of 17α-E2 remain unclear. This form of E2 is more abundant in the brain and adrenal gland compared to the β configuration [54]. Research indicates that 17α-E2 may alleviate metabolic and inflammatory dysfunction in male mice [55]. However, gender dimorphism is observed in its effects, with a more pronounced regulatory role in inflammatory responses seen in males, but not females.

**Figure 1:**
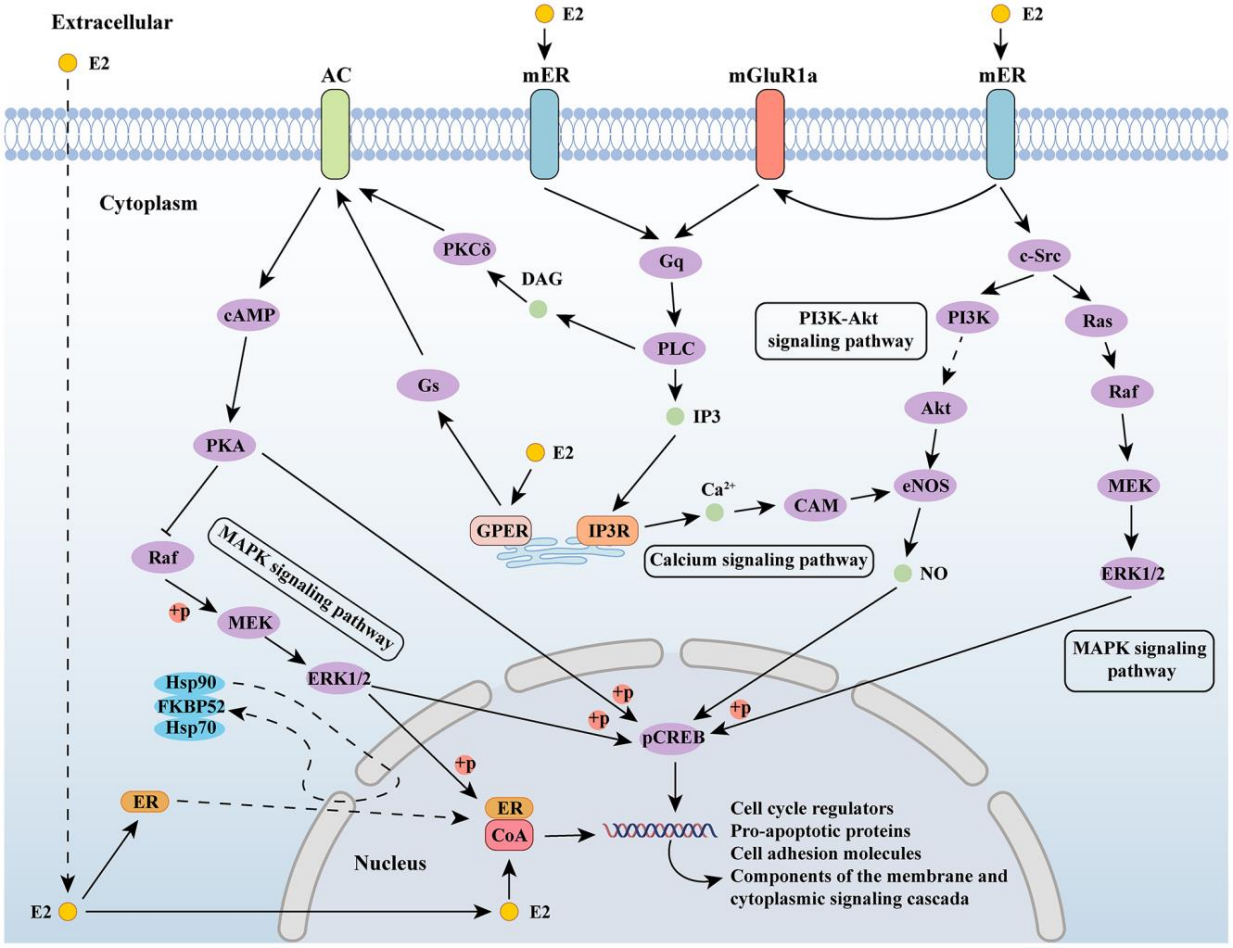
**Estrogen-related signaling pathways.** Abbreviations: E2: estradiol; AC: adenylate cyclase 1; ER: estrogen receptor; mGluR1a: metabotropic glutamate receptor subtype 1a; cAMP: cyclic adenosine monophosphate; PKA: protein kinase A; MEK: mitogen-activated protein kinase; ERK1/2: extracellular signal-regulated kinase 1/2; Gs: guanine nucleotide-binding proteins; GPER: G protein-coupled estrogen receptor; PKCδ: protein kinase C delta; DAG: diacylglycerol; PLC: Portland Limestone Cement; IP3: inositol 1,4,5-trisphosphate; IP3R: IP3 receptor; CAM: crassulacean acid metabolism; PI3K: phosphoinositide 3-kinase; eNOS: endothelial nitric oxide synthase; c-Src: cellular Src; pCREB: phosphorylated cyclic AMP response element binding; CoA: nuclear receptor coactivator.

Estrogens exert their effects by binding to specific receptors and activating signaling pathways. As a steroid hormone, estrogen can enter the plasma membrane to bind to ER in cells and interact with membrane bound estrogen receptors, including G protein-coupled estrogen receptor (GPER) and membrane bound ER (mER), thereby activating intracellular signaling cascades. The signal transduction of estrogen binding to its receptor can be divided into genomic and non-genomic. The signal transduction of the genome involves the binding of estrogen and receptors to form a complex, which migrates to the nucleus and directly acts on the DNA sequence; non-genomic signaling refers to the interaction between estrogen and receptors, which indirectly regulates gene expression through various signaling cascades within cells [56]. Estradiol activates the MAPK signaling cascade through c-Src in the neocortex and regulates gene expression through the mitogen-activated protein kinase (MAPK)/extracellular-signal regulated kinase (ERK) signaling pathway [57, 58]. In hypothalamic neurons, estrogen mediated phosphoinositide 3-kinase/protein kinase B (PI3K/Akt) signaling has also been confirmed [59]. In the central nervous system, voltage-gated calcium channels (VGCCs) interact with estrogen/estrogen receptors [60], and estrogen’s regulation of calcium signaling is also an important component of its intracellular signaling cascade [61] (Figure 1).

**Figure 2:**
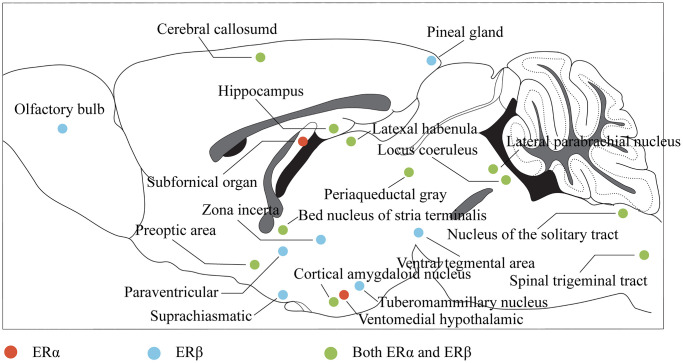
**Distribution of estrogen receptors in the brain.** The color represents the projection of the corresponding brain region in the sagittal plane of the brain, where red indicates that the region mainly expresses ERα, blue indicates that the region mainly expresses ERβ, and green indicates that the region expresses both ERα and ERβ.

Nuclear estrogen receptors, belonging to the nuclear receptor superfamily, form complexes with DNA-binding transcription factors and bind to estrogen, including alpha and beta subtypes. In the central nervous system, estrogen affects cognitive function by binding to estrogen receptor alpha (ERα) and ERβ and regulating the neuroendocrine response through complex signaling pathways. ERα and ERβ are widely distributed throughout the brain and spinal cord [62] (Figure 2), with significant expression in regions crucial for learning, memory, and cognition, such as the PFC, HP, and amygdala (AMY), and the expression of Erα in these regions is greatly increased, while ERβ is decreased in response to stress stimulation [63]. Similarly, stress stimulation combined with ovariectomy significantly reduced ERβ expression in the PFC and hypothalamus [64], suggesting a potential role in coping with anxiety and depression. Additionally, the membrane-bound GPER is widely distributed in the central nervous system and can activate a variety of signal transduction pathways [65]. Studies have shown that ERβ, rather than ERα, plays a critical role in mediating estrogen’s protective effects against depression, while GPER activation also contributes to these effects [38]. Understanding the distribution and function of estrogen receptors in the central nervous system provides insights into the neuroendocrine mechanisms underlying the etiology and treatment of depression.

## Estrogen and depression

### Estrogen and neuroinflammation

Neuroinflammation is a key factor in the pathogenesis of depression, with stress-induced activation of microglia in certain brain structures (such as the HP and AMY) leading to the M1-type phenotype. This activation stimulates the production of chemokines and cytokines (such as interleukin-1beta (IL-1β), interleukin 6 (IL-6), and tumour necrosis factor-alpha (TNF-α)) via the NOD-like receptor family pyrin domain containing 3 (NLRP3) inflammasome pathway. However, M2 microglia can prevent nerve damage caused by M1-type microglia. Polarization of M1 microglia promotes the transformation of A1 but not A2 astrocytes, further contributing to neuroinflammation [66]. Estrogen is believed to have a regulatory effect on glial inflammatory response and may improve depressive behavior by attenuating neuroinflammation. Recent research by Ying Yang suggests that the phytoestrogen Formononetin, which exhibits estrogen-like effects, can reverse the polarization of M1 microglia while promoting the polarization of M2 microglia [67]. Additionally, Soy isoflavones have been found to ameliorate depression-like behaviors by inhibiting hippocampal neuroinflammation and the toll-like receptor 4 (TLR4)/nuclear factor kappa B (NF-κB) signaling pathway [68]. These findings suggest that estrogen and its derivatives may have therapeutic potential in treating depression through their modulation of neuroinflammation.

Ovarianectomy (OVX) is a widely used animal model that mimics estrogen withdrawal-induced depression-like behaviors. This model has been shown to induce neuronal loss, apoptosis, and synaptic dysfunction in brain regions such as the prefrontal cortex (PFC), HP, hypothalamus, and AMY in rats [69]. Additionally, OVX has been associated with a decrease in serum CORT levels and the activation of microglia in the PFC, as well as up-regulation of pro-inflammatory factors such as IL-1β, IL-6, and TNF-α. Conversely, anti-inflammatory factor arginase 1 (Arg1) and microglial negative regulator CD200 were down-regulated following estrogen withdrawal, indicating a pro-inflammatory response [70]. These findings suggest that OVX-induced estrogen withdrawal may contribute to the development of depression-like behaviors through neuroinflammatory processes in the brain. The decline in estrogen levels has been associated with depression-like behaviors, as evidenced by the increased activation of hippocampal microglia, upregulation of pro-inflammatory cytokines, and downregulation of anti-inflammatory factors observed in OVX-induced anxiety and depression-like behavior in rats [71]. Moreover, hippocampal inflammation has been identified as a contributing factor to the development of depression-like behavior in ovariectomized rats [72]. These findings highlight the importance of estrogen in regulating neuroinflammatory responses and its potential therapeutic value in treating depressive disorders.

The estrogen receptor GPER has been implicated in estrogen-mediated neuroprotection. In female OVX rats, the GPER agonist G1 has been shown to inhibit the up-regulation of pro-inflammatory factors, promote the expression of anti-inflammatory factors, and induce microglial transition to the M2 type [73]. Additionally, G1 attenuates the inflammatory response in the CA1 region of the HP by reducing the expression of the microglial marker iba1 and the NLRP3-adaptor protein apoptosis-associated speck-like protein (ASC)-caspase 1 inflammasome, as well as IL-1β activation and downstream NF-κB signaling [74]. Studies have also demonstrated that after OVX, activation of the NLRP3 inflammasome in the HP leads to increased expression of cytokines, whereas estrogen treatment can reverse this response [72]. Furthermore, the activation of the NLRP3 inflammasome has been shown to cause hippocampal inflammation and suppression of ovarian function [75].

**Figure 3:**
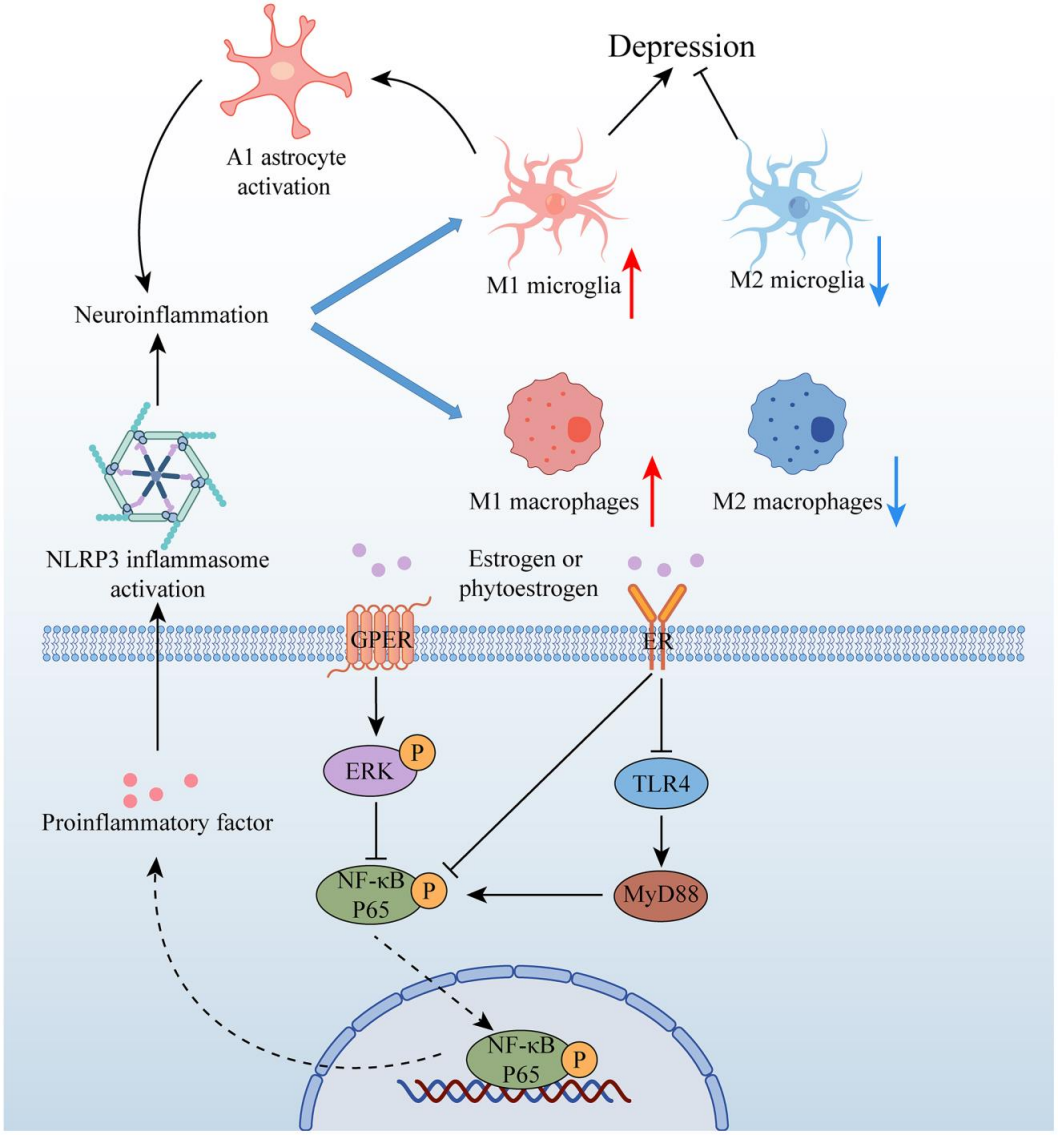
**Relationship between estrogen and neuroinflammation in depression.** Abbreviations: GPER: G protein-coupled estrogen receptor; ER: estrogen receptor; ERK: extracellular signal-regulated kinase; NF-κB P65: nuclear factor kappa-B P65; TLR4: toll-like receptor 4; MyD88.

Estrogen receptors, including ERα and ERβ, are expressed in microglia and astrocytes, and their activation by estrogen triggers signaling pathways such as PI3K/Akt, ERK, or janus kinase/signal transducer and activator of transcription (JAK/STAT), which can lead to neuroprotection and anti-inflammatory effects. Recent studies have shed light on the mechanisms underlying estrogen’s effects on depressive behavior. Specifically, E2 can activate the ERα/sirtuin 1 (SIRT1)/NF-κB signaling pathway, leading to an improvement in depression-like behavior [76]. Furthermore, E2 can inhibit NF-κB activation by binding to ERα in microglia and activating PI3K [77]. In addition, Zhang et al. showed that E2 treatment can alleviate depression-like behavior induced by ovariectomy by upregulating ERα and ERβ in the HP and activating the 5′-Adenosine monophosphate-activated protein kinase (AMPK)/NF-κB signaling pathway [78]. These findings suggest that estrogen’s effects on depression are mediated by its activation of specific signaling pathways, including ERα/SIRT1/NF-κB and AMPK/NF-κB, in microglia and astrocytes. Understanding the molecular mechanisms underlying estrogen’s effects on depression may provide insights into the development of novel treatments for depression (Figure 3).

Clinical studies have proved that the increase of inflammation and vascular dysfunction is one of the important mechanisms of the pathogenesis of depression, and the integrity of the blood-brain barrier (BBB) is an important factor in maintaining the function of the central nervous system [79]. Depression-induced elevation of inflammatory factors is often accompanied by disruption of the integrity of the blood-brain barrier, and in contrast, the neuroprotective effects of estrogen are also mediated by the maintenance of the blood-brain barrier [80, 81]. ERα and ERβ play an important role in the maintenance of BBB integrity by estrogen. The research of Saleh Zahedi Asl proved that both ERα and ERβ agonists can inhibit BBB leakage, and ERα agonists may play a more important role [80]. Vascular pericyte migration is a hallmark of BBB disruption following injury, infection, and inflammation, and TNF-α-induced migration of human brain vascular pericytes was blocked by E2, while ERα and ERβ agonists were found to have similar effects [82].

### Estrogen and neuroendocrine

The HPA axis is a complex neuroendocrine pathway that plays a crucial role in regulating various physiological activities, such as the inflammatory response [83], energy metabolism [84], circadian rhythm [85], and hormonal programming [86], to maintain body homeostasis. In response to external environmental stimuli, the HPA axis is activated, leading to the release of glucocorticoids and other stress hormones that modulate physiological responses. Dysfunction of the HPA axis has been associated with a range of diseases, including depression, anxiety, metabolic disorders, and immune disorders. Understanding the complex interplay between the HPA axis and other physiological systems is critical for the development of effective interventions for various diseases. When the body is exposed to stress, the HPA axis is activated, leading to the secretion of regulatory factors from the hypothalamus. These signals are received by the pituitary gland, which responds by releasing stored or synthesized hormones [87]. The adrenal gland then captures various regulatory signals from the pituitary gland and responds accordingly, secreting glucocorticoids that cause the level of circulating glucocorticoids to rise. Corticotropin-releasing hormone (CRH) and arginine vasopressin (AVP) secreted by the hypothalamic paraventricular nucleus (PVN) neurons act on the pituitary, and the pituitary corticotropin-releasing hormone receptor 1 (CRHR1) mediates stress initiation. The pituitary produces adrenocorticotrophic hormone (ACTH) to act on the adrenal glands, which secrete glucocorticoids. Negative feedback occurs when the rising levels of glucocorticoids return to the hypothalamus PVN neurons, which helps to restore body balance [86, 88].

Glucocorticoid receptors (GR) and mineralocorticoid receptors (MR) are densely expressed in the PVN of the hypothalamus. Ding and colleagues demonstrated that excess glucocorticoids, produced during an emergency response, can bind to GR and MR, altering their ratio to regulate the secretion of CRH and thus exerting negative feedback regulation [89]. In OVX rats, estrogen treatment decreased the expression of GR and MR in the anterior pituitary, HP, and preoptic area (HPOA) of the hypothalamus [90]. As a result, E2 treatment may have an inhibitory effect on the negative feedback of synthetic glucocorticoids, such as dexamethasone. In the presence of E2, the negative feedback mechanism induced by stress-induced CORT increase is delayed, and E2 treatment results in a loss of glucocorticoid receptor-mediated autoregulation [91].

The HPA axis is known to exhibit significant sex-specific differences in terms of hypothalamic PVN composition, pituitary and adrenal function, and hormone release [92]. Females tend to have larger hypothalamic PVN neurons than males [93], and female rats release ACTH more strongly than males after stress stimulation [94]. In fact, female rats are more responsive to ACTH, and OVX surgery can partially reverse this change [95]. Moreover, the release of cortisol from the adrenal glands is also gender-dimorphic, with female rats exhibiting a more pronounced and longer-lasting increase in cortisol levels in response to stress [96]. Interestingly, cortisol itself also shows significant gender dimorphism, with women having approximately 19% more cortisol than men [97], while men tend to have higher levels of free cortisol relative to women [98]. Studies have also observed age-related sex differences in childhood cortisol, with serum and salivary cortisol levels being higher in boys than girls before the age of 8, but the situation reverses after age 8 [99]. Overall, these findings suggest that sex differences in the HPA axis play a critical role in shaping physiological responses to stress and may have important implications for the development of stress-related disorders in males and females.

The activity of CRH neurons in the hypothalamus is higher than that in men, and the expression level of CRH in the hypothalamus is also higher than that in men [100, 101]. The estrogen receptor ER is widely distributed in the hypothalamus [86, 102], and CRH expression is increased in the PVN brain region of depressed patients, accompanied by increased expression of ERα, which is involved in CRH activation. The neuroendocrine and neuronal changes mediated by neonatal maternal deprivation (MD) and postweaning environmental enrichment (EE) in females are strongly linked to anxiety and depression-like behaviors. Specifically, MD reduced the number of oxytocin (OT) immunoreactive neurons in the hypothalamic PVN and co-regulated the expression of CRH and oxytocin receptor (OTR) in the medial-lateral habenula (LHbM) [103]. Estrogen receptors are believed to play a significant role in regulating HPA axis function. Prior research by Wei and others has shown that estrogen induces the expression of both ERα and GR in white matter [104]. Activation of ERβ can also alleviate anxiety-like behaviors and reduce the CORT and ACTH responses of gonadectomy rats [105]. Furthermore, the anxiety-like behaviors caused by selective activation of GR can be mitigated by ERβ agonists [106]. Recent work by Cora E Smiley has demonstrated that stress exposure leads to a specific increase in ERβ and CRH in the central AMY, and blocking ERβ in the AMY improves depressive-like behavior [107]. These findings suggest a complex interplay between the HPA axis, estrogen receptors, and anxiety and depression-like behaviors.

The hypothalamic-pituitary-gonadal axis (HPG) axis is a hormonal system that regulates the secretion of female ovarian hormones, the most widespread source of endogenous estrogens, through a feedback regulatory mechanism [39]. The hypothalamus regulates the pituitary’s production of luteinizing hormone (LH) and follicle-stimulating hormone by releasing gonadotropin-releasing hormone (GnRH), which are used to regulate the female menstrual cycle and promote the secretion of estrogen. Estrogen, in turn, controls the release of these hormones by regulating secretion from the hypothalamus and pituitary gland. Interestingly, estrogen can also be synthesized within the brain, independent of steroid glands, through the action of aromatase and acute regulator proteins expressed in the HP [108]. These hippocampal-derived estrogens regulate gene transcription by binding to their cognate receptors and are thought to be influenced by substrate availability, neuronal activity, and gonadotropins [109]. Thus, the HPG axis plays a crucial role in regulating estrogen levels and its effects on brain function and behavior. There is sufficient evidence to prove that the HPG axis can be regulated by the HPA axis [110]. As an end product of the HPA axis, glucocorticoids have been shown to inhibit the release of GnRH into the pituitary portal system in the hypothalamic median eminence [111]. Similarly, studies have shown that CRH can stimulate or inhibit the discharge of GnRH neurons, and different CRH receptors and CRH concentrations determine whether GnRH neurons are activated or suppressed [111]. The release of GnRH has two modes: pulsatile and surge. During most of the female reproductive cycle, GnRH is released impulsively, while in the late follicular phase, its release mode switches to a sustained increase or surge for several hours [112, 113]. Research has shown that stress affects the surge of GnRH/LH through multi-level activation of the HPA axis, and the glucocorticoid produced by stress can inhibit the surge of LH [114]. These evidences further demonstrate the interaction between the HGA axis and the HPA axis in response to stress stimuli from sources of stress.

### Estrogen and neuroplasticity

Stress and depression can lead to changes in the number and function of synapses. OVX has been shown to reduce dendritic spine density in the CA1 region of the HP, along with enhancement of long-term potentiation (LTP) [115]. However, E2 replacement can counteract this effect [116, 117]. In rodents, phasic changes in dendritic spines and synaptic density in the HP are mediated by physiological fluctuations in estrogen levels, with E2 levels positively correlating with hippocampal synaptic density during the estrous cycle [118–120]. These findings suggest that estrogen plays an important role in modulating synaptic plasticity in the HP, and may have implications for the development of novel treatments for stress-related disorders.

Glutamatergic synaptic dysfunction is a key hallmark of depression. The effects of E2 on glutamatergic transmission can be divided into rapid and chronic. Studies have shown that long-term E2 exposure increases the number of spines positive for the pre-and postsynaptic protein markers bassoon and postsynaptic density protein 95 (PSD95), thereby reversing spine loss [53]. The rapid effects of estrogen on learning and neuronal plasticity are mediated by the membrane receptor GPER and the nuclear receptors ERα and ERβ. GPER activation increases dendritic spine density in the HP [121], while G-protein-coupled-estrogen-receptor 1 (GPER1) promotes the distal dendritic enrichment of hyperpolarization-activated gated channel 1 (HCN1) in CA1 stratum lacunosum-moleculare (SLM) [122]. GPER activation also significantly increases the response of hippocampal CA1 pyramidal neurons to excitatory input, as demonstrated by lateral intracerebral infusion of the GPER agonist G1 [123]. Although all three estrogen receptors contribute to the rapid effects of E2 on hippocampal synaptic function, GPER1 activation appears to play a major role, as ERα and ERβ activation produce only a small increase in synaptic transmission compared to GPER activation [124].

The modulation of inhibitory GABAergic synapses is a critical aspect of regulating plastic changes in neuronal network activity. Previous studies have shown that the expression of GABA transporter-1 (GAT-1) in the medial preoptic area (MPOA) of OVX rats is reduced, and that estrogen replacement can reverse this effect [125]. However, estrogen appears to regulate GABAergic function in a region-specific manner. For example, E2 up-regulates the expression of the 65-kilodalton isoform of glutamic acid decarboxylase (GAD65) and down-regulates the expression of the 67-kilodalton isoform (GAD67) in the magnocellular preoptic area (MCPOA), while the expression of these two glutamate decarboxylases is reversed in the dorsomedial nucleus (DMN) of the hypothalamus [126]. Activation of ERα, but not ERβ, produces a similar effect to E2 by inducing increased synaptic density in hippocampal neurons [127]. Interestingly, a recent study by Zhang demonstrated that activation of ERβ did not affect glutamatergic excitatory transmission in the PFC of female rodents, but it did activate its bisexual GABAergic transmission [128]. These findings suggest that estrogen may differentially modulate GABAergic and glutamatergic synaptic transmission in different brain regions. Overall, these results highlight the complex and region-specific effects of estrogen on inhibitory GABAergic synapses in the brain.

Brain-derived neurotrophic factor (BDNF) is a crucial mediator of neuronal survival, differentiation, and synaptic plasticity. BDNF promotes LTP and enhances salient responses [129], and plays a key role in the antidepressant effect of ketamine [16]. Similarly, estrogen and BDNF have been shown to have overlapping effects on neuronal protection, dendritic spine remodeling, and neurogenesis [130]. E2 exerts its antidepressant effect through the activation of cyclic AMP response-element binding protein (CREB) and BDNF/tropomyosin-related kinase B (TrkB) signaling pathways [131]. In addition, E2 exposure has been shown to upregulate BDNF expression in the PFC of OVX-stressed mice, leading to a reduction in hopelessness and anhedonia [132]. These findings suggest that estrogen and BDNF may work in tandem to promote neuronal plasticity and alleviate depression, making them promising targets for future antidepressant therapies.

Deletion of ERβ, but not ERα, leads to a decrease in BDNF and its receptor TrkB in the HP and PFC of female rodents, while leaving levels unchanged in the cortex and hypothalamus. Interestingly, activation of ERβ can reverse this reduction and even upregulate the expression of major synaptic vesicle proteins p38, vesicle-associated membrane protein 2 (VAMP2), and PSD95 in hippocampal neurons [133]. Inhibitory neurons with ERβ project to other GABAergic neurons that lack nuclear estrogen receptors, and these inhibitory neurons innervate excitatory cells that express BDNF. Additionally, high estrogen levels have been shown to reduce cortical GABA levels, which may release the inhibition of BDNF-expressing neurons [134]. Overall, these findings suggest that ERβ plays a critical role in modulating BDNF expression and synaptic plasticity in specific brain regions, highlighting the importance of understanding the differential effects of estrogen receptors in the brain.

Neurons function within a complex network of connections and the plasticity of neuronal connections is a fundamental feature of the adult brain. The regulation of emotions related to stress and anxiety, as well as the development of depression, are known to involve multiple brain regions, and the role of gender differences in the regulation of neural circuits cannot be ignored [135]. While the locus coeruleus-anterior cingulate cortex (LC-ACC) circuit has been shown to induce pain-related depression in males, its effects in females remain poorly defined [136]. Therefore, investigating estrogen-dependent stress-related neural circuits may be critical for understanding the sexual dimorphism of depression. Research conducted by Tianyao Shi has shown that the anterior insular cortex- basolateral amygdala (AIC-BLA) circuit regulates decision-making behavior related to stress, and estrogens in the AIC may significantly contribute to the sexual dimorphism of stress-induced decision-making behaviors [137]. As a neurosteroid, estrogen is involved in the regulation of LTP and long-term depression (LTD) of synaptic transmission. Zhang found a positive bidirectional association between depression and aggression [138]. Stefanos Stagkourakis demonstrated that LTP at the amygdalohippocampal area- estrogen receptor 1-expressing (AHiPM-Esr1) neurons in the ventrolateral subdivision of the ventromedial hypothalamus (VMHvl), an excitatory synapse, could be induced by aggressive training, and this enhancement of LTP promoted the behavioral effects of aggressive training [139]. Overall, exploring estrogen-dependent stress-related neural circuits may be a key factor in understanding the sexual dimorphism of depression and other related behaviors.

## Estrogen for depression

Estrogen is widely used in the treatment of perimenopausal depression in women, either directly or indirectly. Phytoestrogens such as genistein have been suggested as alternatives to estrogen therapy due to their potential antidepressant and anxiolytic effects in both animal and human studies [140–142]. However, recent clinical trials have shown that the phytoestrogen rimostil and the estrogen receptor modulator raloxifene are not ideal treatments for female psychotic major depression, with transdermal E2 therapy being more effective. While the estrogen-like compounds showed an improvement in depression scores, no significant difference was observed [143]. These findings suggest that while estrogen therapy may be effective in treating depression in women, the choice of treatment should be carefully considered based on the individual’s specific condition and medical history.

Estrogen has been shown to enhance the effects of various antidepressant drugs, such as fluoxetine, venlafaxine, and desipramine [144]. However, caution must be exercised when combining estrogen with imipramine, as it can cause symptoms of toxicity similar to an overdose of imipramine. Moreover, adding estrogen to antidepressant therapy can induce manic symptoms in patients [145]. The therapeutic effect of various drugs on perimenopausal depression is mediated through estrogen and its receptors. In non-human primates, administration of the traditional serotonin reuptake inhibitor, S-citalopram, significantly increased estrogen levels, possibly by modulating CRH levels [146]. Further research is needed to understand the complex interactions between estrogen and various antidepressant drugs, and to develop safe and effective treatment strategies for perimenopausal depression.

The effects of fluoxetine on estrogen action have been investigated in several studies. *In vitro* studies have shown that fluoxetine up-regulates the expression of E2 and down-regulates ERα and ERβ, without affecting aromatase [147, 148]. However, recent research by Lei and colleagues has demonstrated that low concentrations of fluoxetine upregulate the expression of ERα, exhibiting estrogen-like effects [149]. The interaction between fluoxetine and estrogen receptors appears to be dual-natured, with weak estrogenic effects at low concentrations and antiestrogenic effects at high concentrations [150]. In the feto-placental unit co-culture model, fluoxetine did not affect estrogen secretion, but its metabolite norfluoxetine decreased estrogen secretion [151]. Studies in teleosts have shown that fluoxetine exposure causes a decrease in circulating E2 and an increase in ovarian aromatase mRNA expression in female goldfish, with sex-specific disruption of the reproductive endocrine axis [152]. In rodents, co-administration of fluoxetine with E2 produces favorable antidepressant effects in OVX rats by promoting neurogenesis and synaptic plasticity [153]. However, fluoxetine administration had no significant effect on serum E2 levels in female rats [154]. In humans, fetal plasma estrogen levels are not affected by maternal fluoxetine exposure during pregnancy and lactation [155]. Overall, the effects of fluoxetine on estrogen action appear to be complex and depend on various factors, including concentration, duration of exposure, and the species studied. Further research is needed to fully understand the mechanisms underlying these effects.

In a recent study by Zhang and colleagues, the potential therapeutic effects of Erxian Decoction (EXD), a traditional Chinese medicine, on perimenopausal depression were investigated. The researchers found that EXD treatment upregulated estrogen receptors in the uterus and adrenal glands, mitigated estrogen deficiency, and increased the expression of BDNF in the HP, leading to the alleviation of perimenopausal depression [156]. Another traditional Chinese medicine, xiaochaihutang, was also found to have promising antidepressant effects. Through the normalization of the HPA/hypothalamic-pituitary-ovarian (HPO) axis and the restoration of ERβ expression in the PFC, xiaochaihutang was able to ameliorate depression symptoms in ovariectomized rats [64]. Taken together, these findings suggest that traditional Chinese medicines may provide a valuable alternative or complementary therapy for perimenopausal depression by targeting estrogen receptors and the HPA/HPO axis. Further studies are needed to elucidate the underlying mechanisms and potential side effects of these treatments.

Extensive research has highlighted the crucial role of calorie restriction in ameliorating the central nervous system (CNS) lesions. This effect is believed to work synergistically with estrogen, as 48-hour and 1-hour fasting have been shown to trigger ER immune responses in the hypothalamic PVN, periventricular nucleus (PeVN), and nucleus of solitary tract (NTS) regions of OVX rats, resulting in an increase in cells [157]. In patients with Alzheimer’s disease, intermittent fasting has been found to significantly alleviate estrogen deficiency and cognitive dysfunction, potentially via the activation of multiple signaling pathways and neuroprotective effects [158]. Moreover, calorie restriction has shown significant efficacy in treating depression. Acute fasting and E2 have been shown to synergistically activate CREB-BDNF signaling in the PFC and HP, resulting in antidepressant-like effects [159]. Intermittent fasting is also believed to improve cognitive impairment and memory loss caused by estrogen deficiency, cortisol dysregulation, and dyslipidemia [158]. The differential effect of fasting on ERα expression in the pituitary, depending on the levels of estrogen, suggests a complex interplay between metabolic signaling and ovarian hormones [10].

## CONCLUSION

Estrogen and its receptors have been implicated in various hypotheses concerning the pathogenesis of depression, particularly in female depression, making them a central focus of clinical treatment. Adolescent stress can induce abnormal activation and development of the HPA and HPG axes, as well as activate inflammatory signals to induce depression [160]. Estradiol in adolescent women can prevent increased stress-induced neuroinflammation. Similarly, endotoxin, as a common stimulus, can stimulate the release of inflammatory factors from the peripheral immune system, thereby inducing abnormal activation of the HPA axis. This HPA axis response has significant gender differences [161]. The study by Adzic et al. demonstrated that lipopolysaccharide (LPS) induced depressive behavior in women is associated with an increase in hypothalamic CRH and a decrease in BDNF [162]. These evidences all demonstrate that estrogen improves depressive-like behavior through synergistic regulation of HPA axis, neuroinflammation, and neural plasticity, rather than isolated regulation. The gender dimorphism of depression highlights the significance of investigating the direct and indirect effects of estrogen on the mechanisms of depression. Alterations in estrogen levels are accompanied by changes in estrogen receptor expression in different brain regions, regulating estrogen synthesis in both the brain and gonads. The binding of estrogen to receptors mediates the biological function of estrogen in the brain, regulating cognition and mood through a variety of mechanisms such as neuroinflammation, neuroendocrine function, and neural plasticity. Clinical drug treatments for depression often involve changes in estrogen, highlighting the potential for estrogen in the treatment of depression. Furthermore, calorie restriction has been shown to have a positive effect on depression as a safe, side-effect-free and simple treatment. The synergistic effect of calorie restriction on estrogen provides a new physical form for the treatment of depression in women. However, the underlying cellular and molecular mechanisms require further exploration, and the potential risks of this approach need to be elucidated. Overall, a better understanding of the role of estrogen in depression may lead to the development of more effective and personalized treatment methods for depression, especially in female patients. The existing research on the treatment of depression with estrogen mostly focuses on a single mechanism, but the pathogenesis of depression is complex and interconnected, rather than an island. By coordinating multiple mechanisms and focusing on the stable regulation of estrogen levels, it may become a potential and more stable treatment plan for female depression.

### Data availability statement

Data sharing is not applicable to this article as no new data were created or analyzed in this study.
